# General Order in Reference to Sanitary Precautions

**Published:** 1862-06

**Authors:** D. Hunter, Chas. G. Halpine

**Affiliations:** Headquarters Department of the South, Hilton Head, Port Royal, S. C.; Assistant Adjutant General; Headquarters Department of the South, Hilton Head, Port Royal, S. C.


### GENERAL ORDER IN REFERENCE TO SANITARY PRECAUTIONS.

### GENERAL ORDER—NO 5.

*Headquarters Department of the South,*
*Hilton Head, Port Royal, S. C. April* 7., 1862.

1. The Major General commanding desires to call the attention of the officers and men in this department to the paramount necessity of observing rules for the preservation of health during the warm months upon which we have now entered. There is less to be apprehended from battle than from disease, the records of all campaigns in climates such as this showing many more victims to the neglect of sanitary precautions than to the skill, endurance, or courage of the enemy. With proper care exercised, and certain simple rules of hygeine observed, the hardy soldiers of the Union, inured to toil and fortified by habits of industry, temperance, and cleanliness, have nothing to fear from the climate of the department in which it is their privilege to serve. During our war with Mexico the soldiers of New England, the Northwestern and Middle States, and the adopted citizens serving in our army, suffered far less from the diseases incident to a semi-tropical climate than the soldiers from the States embraced in this department. Though not so well accustomed to excessive heat, their physical energies had been more fully developed by habits of steady industry, and their constitutions presented greater natural obstacles to the inroads of malaria. Anxious that the men of his command may be preserved in the full enjoyment of health to the service of the Union, and that only those who can leave behind them the proud epitaph of having fallen on the battle-field in defence of their country shall fail to return to their homes and avocations on the termination of this unholy rebellion, the Major-General commanding, in conformity with the excellent advice of Surgeon George E. Cooper, United States Army, Medical Director of the Department, hereby establishes the following rules for the sanitary government of all the troops at present serving, or hereafter to serve, in Georgia, South Carolina, and Florida, and will hold all officers having the charge of camps or post, to a strict responsibility for their enforcement.

II. Care will be taken in the selection of camping grounds to avoid as much as possible the vicinity of malarious morasses or swamps: and the tents, in so far as practicable, are to be faced to the south. Each camp will be thoroughly policed twice each day, morning and evening, and all garbage or refuse matter will be collected and buried in the sinks.

III. Each tent will be screened or covered at the top and half-way down the sides with an arbor of brushwood or palm leaves, and shall be floored, whenever lumber can be procured, at an elevation of about three inches from the ground. When this cannot be done, each soldier will have a bunk raised eighteen inches from the ground on side poles, supported by forked sticks. All Quartermasters, to the extent of their ability, will furnish barrel staves to be placed across these side poles, and will issue the necessary lumber on receipt of proper requisitions.

IV. Tents will be struck at least three times each week, and every article of bedding and clothing taken out and aired, the flooring and bunks to be thoroughly cleansed before the tents are re-erected. On the days on which the tents are not struck the sides will be raised and kept raised for the purpose of ventilation; and during the nights free ventilation will be secured by having the centre seen in rear of the tent opened for the space of two feet, and kept open by the insertion of a forked stick. An officer of each company will inspect the tents of his men nightly, except during stormy weather, to see that this important provision is carried out.

V. Sinks of the proper size, screened with brushwood or palmetto branches, shall be sunk at suitable distances on different sides of each camp, and the bottoms of these will be covered each morning with a layer of sand or clay about a foot thick. It will be the duty of the camp police to see that only the sinks on the lee side of the camp are used.

VI. Fresh meat is to be issued as often as practicable, and commanding officers, while near the seacoast or any pieces of water in which fish exist, should encourage such of their men as are off duty or not otherwise employed, to fish during the cool hours of the morning and evening, not later than nine A.M. in the morning, and not earlier than six P.M. in the evening. In a scarcity of fresh meat those troops in the most exposed and unhealthy situations are to be first served—the troops stationed in the batteries on the Savannah river, for instance; and to all troops so placed a large share of vegetables, in addition to the ordinary rations, should be sent.

VII. Vegetables, fresh or prepared, must be issued frequently to all the troops, and an extra issue of coffee furnished to the men on guard during the night, just previous to their being marched to their respective stations. The Chief Commissary of Department will see that the estimates and requisitions necessary to fulfil these requirements are forwarded to the Commissary General without delay, and will report to these head-quarters any failure of brigade or regimental commissaries to make due requisition for the supplies of the troops under their charge, in conformity with the terms of this order.

VIII. Breakfast will be ready for the men as soon as they leave their tents, which must not be until after sunrise. Except when immediately in face of the enemy, or when especially ordered by the commanding officer, reveille will not be sounded until half an hour after sunrise, by which time the sun's heat will have absorbed the miasma of the night dews. All the men will be furnished with straw hats, and will be required to bathe or wash themselves thoroughly at least twice each week, and change their underclothing once a week, or oftener if practicable. The hair and beard will be kept closely trimmed; and sentry boxes of lumber or small shade arbors of brushwood will be erected at all points where sentries are permanently stationed. All soldiers on night picket or sentry duty will be provided with india rubber ponchos.

IX. The proper cooking of provisions is a matter of great importance, more especially in this climate, but has not yet received from a majority of the officers in our volunteer service that attention which is paid to it in the regular army of the United States, and by the armies of Europe. Hereafter, an officer of each company will be detailed to superintend the cooking of provisions, taking care that all food prepared for the soldiers is sufficiently cooked, and that the meats are boiled or roasted, not fried. With a little care on this point, and the advantages both to health and comfort of good cooking explained to the men, much good may be effected.

X. All soldiers on duty in districts especially malarious, or on avoidable fatigue duty during the hot hours of the day, should be given quinine in prophylactic doses, each dose combined with half a gill of whiskey, every night and morning. The certificates of regimental surgeons will be requisite to cover such issues.

Officers of the medical staff will see that the provisions of this order are complied with, and will promptly report any failure or neglect to the senior officers of the commands they are serving with, and to the medical director of this department.

By command of

Major-General D. HUNTER.

CHAS. G. HALPINE, Assistant Adjutant General.

